# The *Gustavus* Gene Can Regulate the Fecundity of the Green Peach Aphid, *Myzus persicae* (Sulzer)

**DOI:** 10.3389/fphys.2020.596392

**Published:** 2021-01-12

**Authors:** Yang Gao, Ruifan Ren, Jing Peng, Dongwei Wang, Xiaobin Shi, Limin Zheng, Zhuo Zhang, Chunhui Zhu, Yong Liu, Liangying Dai, Deyong Zhang

**Affiliations:** ^1^College of Plant Protection, Hunan Agricultural University, Changsha, China; ^2^Hunan Plant Protection Institute, Hunan Academy of Agricultural Science, Changsha, China; ^3^Long Ping Branch, Graduate School of Hunan University, Changsha, China

**Keywords:** *Gustavus* gene, *Myzus persicae* (Sulzer), fecundity, RNA interference, potato virus Y

## Abstract

*Myzus persicae* (Sulzer), commonly known as the green peach aphid, is a notorious pest that causes substantial losses to a range of crops and can transmit several plant viruses, including potato virus Y (PVY). Chemical insecticides provide only partial control of this pest and their use is not environmentally sustainable. In recent years, many genes related to growth, development, and reproduction have been used as targets for pest control. These include *Gustavus* (*Gus*), a highly conserved gene that has been reported to play an essential part in the genesis of germline cells and, hence, in fecundity in the model insect *Drosophila melanogaster*. We hypothesized that the *Gustavus* (*Gus*) gene was a potential target that could be used to regulate the *M. persicae* population. In this study, we report the first investigation of an ortholog of *Gus* in *M. persicae*, designated *MpGus*, and describe its role in the fecundity of this insect. First, we identified the *MpGus* mRNA sequence in the *M. persicae* transcriptome database, verified its identity with reverse transcription-polymerase chain reaction (RT-PCR), and then evaluated the transcription levels of *MpGus* in *M. persicae* nymphs of different instars and tissues with real-time quantitative PCR (RT-qPCR). To investigate its role in regulating the fecundity of *M. persicae*, we used RNA interference (RNAi) to silence the expression of *MpGus* in adult insects; this resulted in a significant reduction in the number of embryos (50.6%, *P* < 0.01) and newborn nymphs (55.7%, *P* < 0.01) in the treated aphids compared with controls. Interestingly, *MpGus* was also significantly downregulated in aphids fed on tobacco plants that had been pre-infected with PVY^*N*^, concomitant with a significant reduction (34.1%, *P* < 0.01) in *M. persicae* fecundity. Collectively, these data highlight the important role of *MpGus* in regulating fecundity in *M. persicae* and indicate that *MpGus* is a promising RNAi target gene for control of this pest species.

## Introduction

*Myzus persicae* (Sulzer) (Hemiptera: Aphididae) is a pest that can infest crops all over the world (van Emden and Harrington, 2017). This aphid is known to extract nutrients from the phloem and xylem of more than 400 plant species in over 50 families (Smith, 1926; van Emden et al., 1969, van Emden and Harrington, 2017; Weber, 1986; Spiller et al., 1990; Bernays and Chapman, 1994; Gupta, 2001; Chen et al., 2020), including both row crops and ornamentals grown in greenhouses. *M. persicae* reduces crop yields by causing systemic perturbations in nutrient allocation, leaf discoloration and necrosis, and leaf and/or fruit deformation (Zhan et al., 2020). Furthermore, *M. persicae* is one of the most effective vectors for plant viruses (Ng and Perry, 2004; Ng and Falk, 2006) and can transmit a large number of economically important viruses in a persistent, semi-persistent, or non-persistent manner (Carmo-Sousa et al., 2014). Under certain conditions, *M. persicae* can produce wings for migration; they can also be transported over long distances by wind and storms. *M. persicae* breeds in a highly prolific manner, making these particular aphids very difficult to control. Currently, the main form of control for *M. persicae* is the application of chemical insecticides. However, the efficacy of insecticides can be limited by over-application and the rapid development of resistance (Bass et al., 2014). Furthermore, long-term use of pesticides can cause environmental damage. Consequently, there is a clear need to develop new control strategies that are more selective and have better environmental profiles.

RNA interference (RNAi), mediated by double-stranded RNA (dsRNA), has been shown to be an effective means of controlling a large number of agricultural pests (Upadhyay et al., 2011; Zhang et al., 2015; Niu et al., 2018; Liu et al., 2019; Christiaens et al., 2020). Recent reports have also identified a range of genes that could be used as RNA targets to control aphids. For example, Hou et al. (2018) showed that downregulation of the guanine nucleotide-binding protein G(q) subunit alpha (*Gq*α) gene resulted in a reduction in the molting rate and fecundity of the grain aphid (*Sitobion avenae* F.). Similar results were observed by Ye et al. (2019), who used RNAi to target the chitin synthase gene in pea aphids (*Acyrthosiphon pisum*). In another study, Tariq et al. (2019) demonstrated that knockdown of the voltage-gated sodium channel gene caused an increase in mortality of *M. persicae*. In addition, silencing of the gene that encodes cuticular protein has been shown to result in impaired fecundity in *M. persicae* (Bhatia and Bhattacharya, 2018). A range of methods have been used to deliver dsRNA to pests. For example, Zhao et al. (2018) fed *S. avanae* with transgenic leaves expressing dsRNA targeting the c*hitin synthase 1* gene, and observed a significant reduction in the size of the insect population. Yan et al. (2019) reported that spraying dsRNA on *Aphis glycines* also led to a substantial reduction in the expression levels of target genes. Gui et al. (2020) completely inhibited oviposition in *Leptinotarsa decemlineata* by injecting dsRNA. Most recently, Lü et al. (2020) fed *Henosepilachna vigintioctopunctata* with leaf discs that had been immersed in ds*Hvlwr* solution, causing an increase in mortality. Although RNAi is considered to be a revolutionary strategy for pest control, the efficacy of this technique is highly dependent on the identification of target genes that are responsive to RNAi-mediated mRNA degradation and on the efficiency of delivery of dsRNA to the target. Genes involved in the reproductive cycle of pests are likely to represent highly promising targets for RNAi.

Gustavus (Gus) is a protein that was first identified in the fruit fly (*Drosophila melanogaster*) as a positive regulator of insect spawning (Styhler et al., 2002; Gustafson et al., 2011). The Gus protein possesses both a sp1A/ryanodine receptor (SPRY) domain and a suppressor of cytokine signaling (SOCS) box, so it belongs to the SPRY domain-containing SOCS box (Spsb) family of proteins (Styhler et al., 2002; Gustafson et al., 2011). Previous studies have shown that Gus interacts with VASA, a protein in *D. melanogaster* that has key roles in the specialized translational activity and localization of the pole plasm, and in the specification of germ cells. Mutations in the *Gus* gene have been reported to cause female sterility in the fruit fly (Styhler et al., 2002). Collectively, these studies indicate that the Gus protein has critical roles in the reproductive success of a diverse number of arthropods.

The rate of reproduction, also known as fecundity, is a common insect trait that can be frequently modified by the plant viruses they transmit (Ziebell et al., 2011; Mauck et al., 2014; Casteel et al., 2015). For example, previous studies showed that potato virus Y (PVY; genus *Potyvirus*; family *Potyviridae*), a virus known to be transmitted by *M. persicae* in a non-persistent manner, affected the fecundity of *M. persicae* (Srinivasan and Alvarez, 2007; Valkonen et al., 2007; Cervantes and Alvarez, 2011; Ren et al., 2015; Bak et al., 2017). However, it is not yet known whether the *Gus* gene is involved in the process of PVY affecting aphid fecundity.

Based on these previous findings, we hypothesized that orthologs of the Gus protein may also serve to regulate the fecundity of *M. persicae*, and that the *Gus* gene plays a part in the process of PVY affecting aphid fecundity. To test this hypothesis, we identified *MpGus*, a *Gus* ortholog in *M. persicae*, and then determined the expression profile of *MpGus* in different instars. Next, we used dsRNA-mediated RNAi to silence the expression of *MpGus*. Finally, we fed PVY-infected tobacco plants to *M. persicae* and investigated the changes in *MpGus* expression and fecundity. Our results showed that *MpGus* has a crucial role in the fecundity of *M. persicae*, and that its expression and functionality are regulated by the acquisition of PVY. These findings suggest that *MpGus* could be used as an RNAi target to control *M. persicae*.

## Materials and Methods

### Plant and Insect Care

Tobacco (*Nicotiana tabacum* L. cv. Samsun NN) plants were kept in aphid-proof screen cages in a greenhouse room set to 25 ± 1°C and 75 ± 5% relative humidity, with a 16 h light/8 h dark photoperiod. In order to systemically infect tobacco plants with PVY, we mechanically inoculated plants at the five-true-leaf stage with leaf sap containing the PVY N strain (PVY^*N*^) (Shrestha et al., 2014). Symptoms (veinal necrosis) appeared after 10 days and the infection was further confirmed by reverse transcription-polymerase chain reaction (RT-PCR) assay using primers that were specific to the coat protein (*CP*) gene (PVY-F/R; Table 1; Liu et al., 2010).

**TABLE 1:** Primers used in this study.

Primer sequence	(5′–3′)
*PVY*-F^*a*^	TTCATCTCCATCCATCATAACC
*PVY*-R^*a*^	TACAACTTGCATACGACATAGG
*MpGus-*F_1_	ATGCCAGCAGTGGTTTTGC
*MpGus-*R_1_	TTATCTCCTGTCCCGGTATAATAAG
*MpGus-*F_2_	ATGCCAGCAGTGGTTTTGC
*MpGus-*R_2_	TTATCTCCTGTCCCGGTATAATAAG
*MpGus-*F_3_	ATGAACCGCGATTACCATACTG
*MpGus-*R_3_	TTATCTCCTGTCCCGGTATAATAAG
RT-qPCR-*MpGus*-F	GCAACCTTACAAGCCCGTAG
RT-qPCR-*MpGus*-R	CCAGCCGTGTTTGATTTG
*Mp-actin*-F^*b*^	CGGTTCAAAAACCCAAACCAG
*Mp*-*actin*-R^*b*^	TGGTGATGATTCCGTGTTC
*MP*-*GAPDH*-F^*c*^	TTCTGTTGTTGACTTGAC
*MP*-*GAPDH*-R^*c*^	CTTCATCTTCAGTGTAACC
ds*MpGus*-F	TAATACGACTCACTATAGGGCGCATTAGTAGGCACCAGC
ds*MpGus*-R	TAATACGACTCACTATAGGGCAGCCGTGTTTGATTTGC
ds*GFP*-F	TAATACGACTCACTATAGGGTCAGTGGAGAGGGTGAAGGT
ds*GFP*-R	TAATACGACTCACTATAGGGGTGTGGACAGGTAATGGTTG

*^*a*^Primers for PVY originally reported by Liu et al. (2010).*

*^*b*^Primers for Mp-actin originally reported by Pitino et al. (2011).*

*^*c*^Primers for Mp-GAPDH originally reported by Wang et al. (2019).*

*Underlined section represents the T7 promoter sequence.*

The *M. persicae* individuals used in this study were descendants of a single adult and were reared for more than 10 generations on healthy tobacco plants before being used in our experiments.

### *MpGus* Cloning and Bioinformatics Analysis

Total RNA was extracted from aphids that contain all nymphal and adult stages using TRIzol reagent (Thermo Fisher Scientific, Shanghai, China) and quantified by a NanoDrop spectrophotometer (Model ND-2000, Thermo Fisher Scientific, Waltham, MA, United States). First-strand cDNA was synthesized from 500 ng of total RNA using TransScript All-in-One First-Strand cDNA Synthesis SuperMix for PCR (TransGene Biotech, Beijing, China), in accordance with the manufacturer’s instructions. The resulting cDNA was then used as templates for PCR reactions to amplify the full-length coding sequence of *MpGus* using the primers *MpGus-*F_1–3_/R_1–3_ (Table 1); these primers were designed to the sequence information provided by the *M. persicae* genome assembly (NW_019102926.1). The PCR reaction included 1 μL of KOD-Plus-Neo (Toyobo Life Scientific, Shanghai, China), 5 μL of 10 × PCR buffer for KOD-Plus-Neo, 3 μL of 25 mmol/L MgSO_4_, 5 μL of 2 mmol/L dNTPs, 1 μL of each primer (10 μmol/L), 1 μL of cDNA template, and 33 μL of ultrapure water, in a total volume of 50 μL. The following cycling conditions were used for PCR: 94°C for 2 min, 35 cycles of 15 s at 98°C, 15 s at 58°C, and 90 s at 68°C. The PCR products were purified with an EasyPure PCR Purification Kit (TransGene Biotech, Beijing, China) and then sequenced.

Sequences were analyzed at the nucleotide and protein level using BLAST^1^ Multiple sequence alignments were generated using Clustal Omega^2^. A phylogenetic tree was also constructed using the amino acid sequences of Gus orthologs from 14 different species; for this, we used the neighbor-joining method in the MEGA 7 software (Kumar et al., 2016). The bootstra *p*-values (as percentages) shown at the branch points were calculated from 1,000 replicates^3^.

### The Expression Profile of *MpGus* in Different Instars and Tissues

**FIGURE 1:**
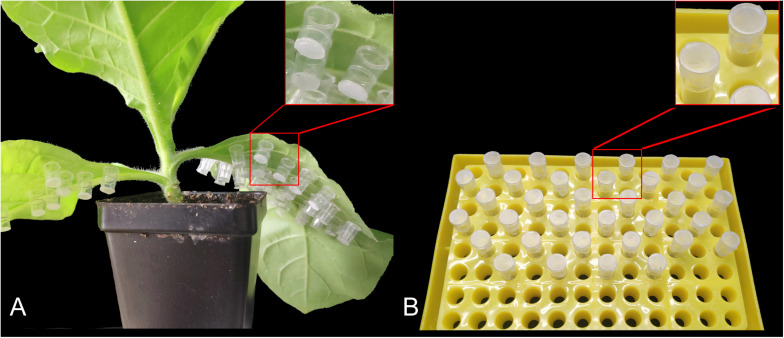
Mini cages used to rear individual *Myzus persicae* adults. **(A)** Mounting of the mini cages on tobacco leaves. **(B)** Mini cages supplied with artificial diets.

To collect the nymphs of different instars, we infested 10 healthy tobacco plants with 10 apterous *M. persicae* adults per plant. These adults were subsequently removed after 24 h; 150 newborns were collected as the first instar nymphs. The rest of the nymphs were covered individually using home-made mini cages. These cages were made from 0.2 mL PCR tubes with lids and the bottom halves removed, followed by sealing of the bottoms with 100-mesh gauze; they could be pasted onto the leaves with starch dextrin (Figure 1A). Over the next few days, we monitored the nymphs for molting activity and collected 80 s instar nymphs, 40 third instar nymphs, and 20 fourth instar nymphs. To collect cephalothorax, leg, and abdomen of apterous adult aphids, we dissected 50 aphids under a stereoscopic microscope (LEICA S8AP0). The collected samples were placed individually in 1.5 mL RNase-free centrifuge tubes, rapidly frozen in liquid nitrogen, and stored at −80°C until expression profiling of *MpGus*.

The expression levels of *MpGus* in different instars and tissues were determined by real-time quantitative PCR (RT-qPCR) with specific primers (RT-qPCR-*MpGus*-F/R; Table 1). The amplification efficiency of these primers was 94.6%. According to Bustin et al. (2009), the relevant information for PCR primer verification is provided in Supplementary Figures S1, S2. RT-qPCR reactions were carried out in a 96-well optical plate in an Analytik Jena AG PCR instrument; the system software was used to normalize and quantify expression data. The target gene was amplified with 2 × TransStar Green qPCR SuperMix UDG (TransGene Biotech, Beijing, China). For each sample, we performed three repetitions of three biologically independent experiments. The relative expression levels of the *MpGus* gene were then calculated using the 2^–△△*Ct*^ method (Livak and Schmittgen, 2001). β*-actin* and *GAPDH* genes from *M. persicae* were used as reference genes to normalize gene expression levels (Table 1; *Mp*-*actin*-F/R, *Mp*-*GAPDH*-F/R).

### RNAi Methodology

Primers were designed to amplify a 442 bp PCR fragment from *MpGus*. Both primers (ds-*MpGus-*F/R) contained the T7 promoter sequence at their 5’ ends (Table 1). The PCR product was purified using an EasyPure^®^, PCR Purification Kit (TransGene Biotech, Beijing, China) and used as a template to synthesize dsRNA targeting *MpGus* (ds*MpGus*) using a MEGAscript^®^, RNAi Kit (Thermo Fisher Scientific, Waltham, MA, United States). Once synthesized, ds*MpGus* was purified and resuspended in nuclease-free water. Next, we verified the molecular weight of ds*MpGus* by 1.5% agarose gel electrophoresis. Concentrations of purified dsRNAs were determined using a NanoDrop spectrophotometer (Model ND-2000, Thermo Fisher Scientific, Waltham, MA, United States). The purified ds*MpGus* was stored at −20°C prior to use. We also used specific primers (ds*GFP-*F/R; Table 1) to synthesize a ds*GFP* control in the same manner.

For the RNAi assays, we added different concentrations of ds*MpGus* or ds*GFP* to an artificial diet, as described previously by Sadeghi et al. (2009). The artificial diet for *M. persicae* was prepared according to the method described by Dadd and Mittler (1966), with slight modifications. Next, we transferred aphids that had begun to reproduce from healthy plants and fed them on an artificial diet containing 250, 500, or 750 ng/μL (final concentration) of dsRNA. In order to minimize degradation of the dsRNA in the artificial diet or insect bodies (Allen and Walker, 2012; Yu et al., 2013), we replaced the diet every 24 h. To analyze the gene suppression of cognate mRNA, we collected 30 insects as one sample from each treatment group after 24 h; these were then flash-frozen in liquid nitrogen, and stored at −80°C in 1.5 mL centrifuge tubes until extraction of total RNA.

To further clarify the *MpGus* transcripts levels at different time points under the effective ds*MpGus* concentration, we treated aphids using the same method as described above with effective concentrations of ds*MpGus*, collected samples at 6, 12, 24, 48, and 72 h, respectively, and subsequently assayed them individually by RT-qPCR. We also measured the effect of dsRNA on aphid mortality. The experiment included 3 groups: H_2_O + artificial diet (CK), ds*GFP* + artificial diet and ds*MpGus* + artificial diet. Each group contained 75 aphids. The number of dead aphids was recorded every 24 h for 5 consecutive days.

### Analysis of the Effects of Tobacco Infected With PVY^*N*^ on *MpGus* Gene Expression

To assess whether PVY^*N*^ could alter the expression levels of *MpGus* in *M. persicae*, we compared *M. persicae* fed on tobacco plants infected with PVY^*N*^ with those fed on mock-inoculated tobacco plants. Aphids that began to reproduce were taken from healthy plants and transferred to PVY^*N*^-infected tobacco. Next, we randomly collected 30 aphids as one sample from the leaves of PVY^*N*^-infected/mock-inoculated tobacco plants after 24, 48, and 72 h. The collected aphids were stored at −80°C in 1.5 mL centrifuge tubes and subsequently assayed individually by RT-qPCR.

### Fecundity Analysis

Next, we attempted to determine the fecundity of *M. persicae* exposed to the dsRNA diet. To do this, we collected 150 adult aphids that were just beginning to reproduce and transferred them individually to separate mini cages (one aphid per mini cage). Individual aphids were then fed with dsRNA; a small piece of parafilm, containing 5 μL artificial diet mixed with ds*MpGus* (final concentration 500 ng/μL) was placed at the top of each mini cage (Figure 1B). Half of these individual aphids (75) were fed with ds*MpGus*; the other half were fed with ds*GFP* as a non-specific dsRNA control. Once, the aphids had been fed for 24 h, we began to count newborn nymphs. All counted nymphs were removed to ensure that only new nymphs were counted in subsequent measurements. Counting sessions were carried out once every 24 h for 5 days.

To determine the fecundity of aphids that had been exposed to PVY^*N*^, the aphids that had been maintained separately on non-viruliferous tobacco were subsequently transferred to a mini cage mounted on viruliferous tobacco plants; we did this when the aphids first began to reproduce. Each mini cage, containing an individual aphid, was mounted on the lower surface of the third to the sixth blade of the plants. A set of 75 adults were prepared for each experimental group. The newborn nymphs in each mini cage were counted once every 24 h, for 5 days. Once counted, nymphs were removed. Aphids maintained on mock-inoculated tobacco were used as controls.

### Quantification of Developing Embryos

Apterous adult aphids treated with ds*GFP* or ds*MpGus* for more than 24 h were transferred to clean slides. Their abdomens were then dissected with a needle under a stereomicroscope (LEICA S8AP0). For each female aphid, we only counted developing embryos with obvious red compound. A total of 15 females were inspected per treatment.

### Data Analysis

Statistical analyses were performed with the SPSS package (version 22.0; SPSS Inc., Chicago, IL, United States). For normally distributed data (Shapiro–Wilk test: *P* > 0.05), we used the independent Student’s *t*-test to compare experimental and control data. Data that did not conform to a normal distribution (Shapiro–Wilk test: *P* ≤ 0.05), were compared using a non-parametric Mann–Whitney *U*-test. The expression levels of *MpGus* in different instars were compared by one-way analysis of variance (ANOVA) and Tukey’s *post hoc* test. The independent Student’s *t*-test was used to compare the expression levels of *MpGus* induced by RNAi and PVY^*N*^ in different groups. The number of newborn nymphs from different treatment groups was compared using the Mann–Whitney *U*-test. Means were compared by the least significant difference test at *P* = 0.05.

## Results

### Identification of *MpGu*s (the Ortholog of *Gus* in *M. persicae*)

**FIGURE 2:**
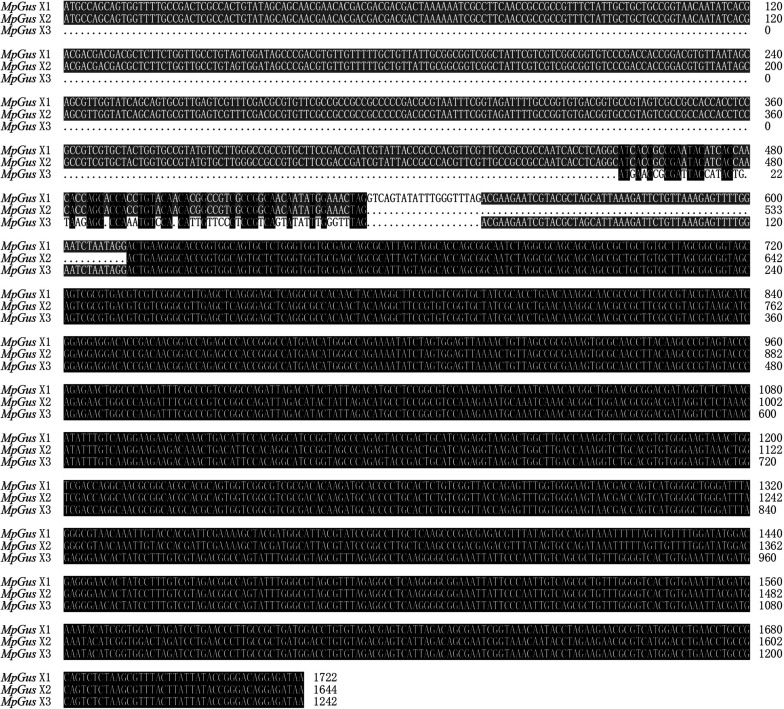
Sequence alignment of three transcriptional variants. Gray indicates that two sequences are consistent; black indicates that three sequences are consistent.

**FIGURE 3:**
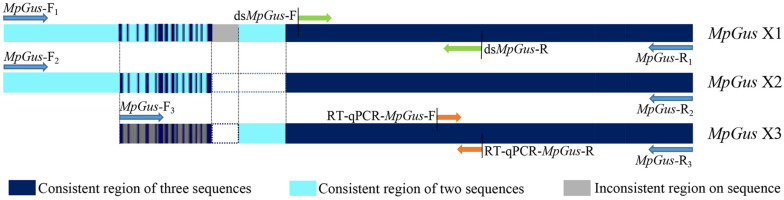
Position information for the primers used in the experiment in the *MpGus* sequence. *MpGus*-F_1–3_/R_1–3_ were amplification primers for the full length sequence of *MpGus* X1, *MpGus* X2, and *MpGus* X3, respectively. ds*MpGus*-F/R were used to prepare ds*MpGus*. RT-qPCR-*MpGus*-F/R were used to detect the expression levels of *MpGus*.

Three cDNA sequences that represent three predicted transcript variants of the same *M. persicae* gene, named *MpGus* X1, *MpGus* X2, and *MpGus* X3, have been annotated as *Gus* in GenBank (term = + Gustavus + Myzus)^4^. We thus presumed that these genes were the *M. persicae* orthologs of *Gus* and designated these genes as *MpGus*. To determine whether all three of the splicing variants were expressed in aphid bodies, we designed three pairs of specific primers and used these in RT-PCR to amplify separate fragments. Each primer pair allowed the amplification of a full-length coding sequence. The amplified sequences of *MpGus* X1, *MpGus* X2, and *MpGus* X3 were identical to XM_022314172.1, XM_022314173.1, and XM_022314174.1 in GenBank, respectively. Although there were differences in the N-terminals of the three sequences, the SPRY and SOCS domain were completely identical (Figure 2). In order to study the function of *MpGus*, ds*MpGus* was prepared, targeting the conserved regions of the three transcriptional variants, and specific primers RT-qPCR-*MpGus*-F/R for RT-qPCR detection were also designed according to the conserved regions (Figure 3).

### The Gus Protein Shows High Levels of Conservation Across a Diverse Range of Species

**FIGURE 4:**
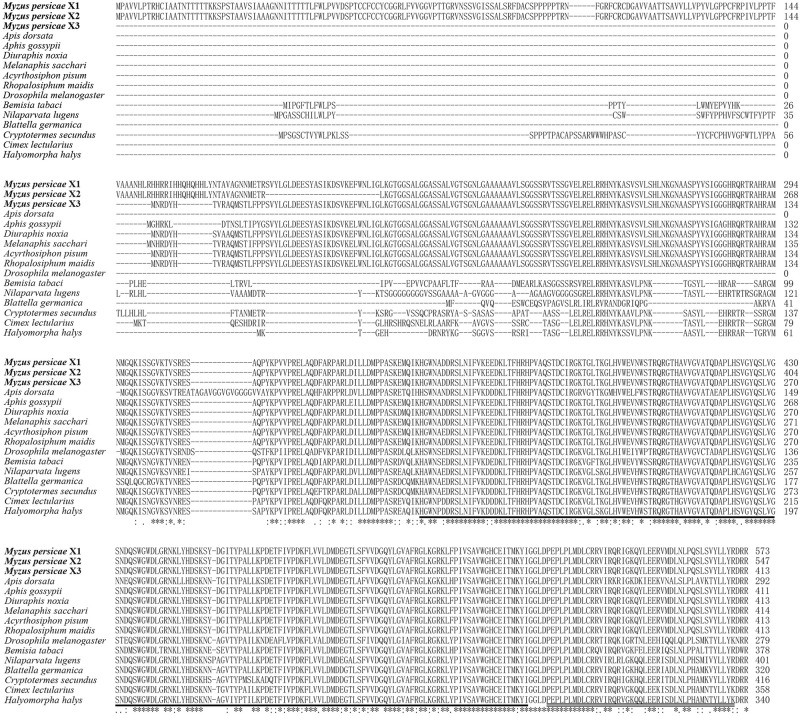
Multiple sequence alignments of *MpGus* with other 13 *Gus* orthologs from diverse species. The GenBank accession numbers of these species are *Myzus persicae* X1 (XP_022169864.1); *Myzus persicae* X2 (XP_022169865.1); *Myzus persicae* X3 (XP_022169866.1); *Aphis gossypii* (XP_027839423.1); *Diuraphis noxia* (XP_015372875.1); *Melanaphis sacchari* (XP_025207865.1); *Acyrthosiphon pisum* (XP_016662132.2); *Rhopalosiphum maidis* (XP_026820642.1); *Apis dorsata* (XP_006616315.1); *Drosophila melanogaster* (sp| A1Z6E0.2|); *Bemisia tabaci* (XP_018912944.1); *Nilaparvata lugens* (XP_022187205.1); *Blattella germanica* (PSN58391.1); Cryptotermes secundus (XP_023706529.1); *Cimex lectularius* (XP_014262520.1);, and *Halyomorpha halys* (XP_014272688.1). Highly conserved amino acid residues across all of the species compared are annotated by *. The SPRY domain is underlined, and the SOCS box is marked by double underscores.

**FIGURE 5:**
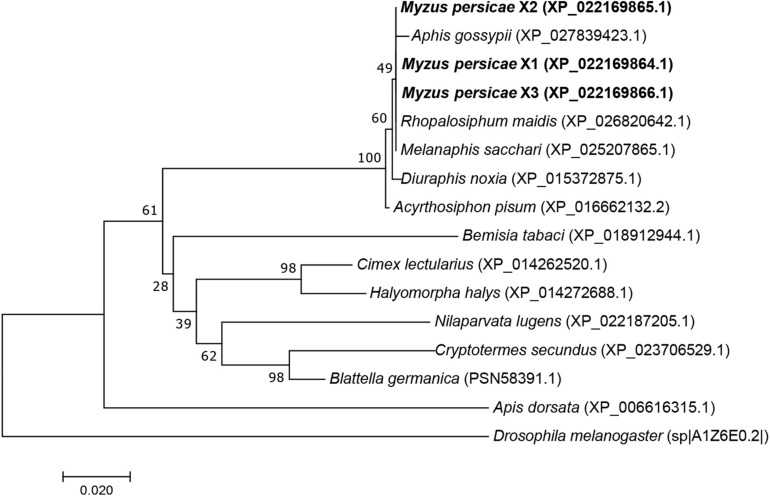
A phylogenetic tree of Gus proteins from different organisms based on the comparison of amino acid sequences. The proportion (%) of replicate trees are shown next to the branches. MpGus is shown in bold. *Drosophila melanogaster* was included as an outgroup.

The MpGus protein contains a conserved SPRY domain and the SOCS box that is shared by all proteins in the Spsb protein family. Compared with the Gus orthologs of other arthropods, the amino acid sequence identity ranged from 77.4 to 99.8% (Figure 4). Further phylogenetic analysis showed that the MpGus protein clustered closely with its orthologs in other aphid species, including *Aphis gossypii*, *Rhopalosiphum maidis*, *Melanaphis sacchari*, *Diuraphis noxia*, and *A. pisum*. As expected, the MpGus protein was more distantly related to the Gus orthologs of *Bemisia tabaci*, *D. melanogaster*, and *Apis dorsata* (Figure 5). Nevertheless, regions representing the SPRY and SOCS domains were highly conserved across all of the Gus orthologs we compared.

### Expression Levels of *MpGus* at Different Developmental Stages of Nymphs and in Different Tissues

**FIGURE 6:**
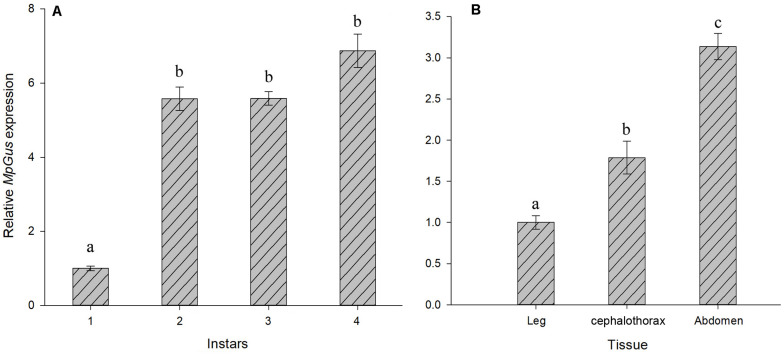
Temporospatial expression profiles of *MpGus*. **(A)** Relative expression of *MpGus* in different instars, as measured by RT-qPCR. **(B)** Relative expression of *MpGus* in different instars, as measured by RT-qPCR. Each data point represents the mean of three independent samples, with three repetitions per sample. mRNA expression values were normalized to reference genes (β*-actin* and *GAPDH*). Data represent mean ± standard error (SE), *n* = 3, and different letters indicate statistically significant differences at *P* < 0.05.

Next, we used RT-qPCR to investigate changes in the expression of *MpGus* during the development of different nymph stages. The mRNA levels of *MpGus* were lowest in first instar nymphs but increased rapidly as nymphs developed from the first to the second instar stage [*F*_(1,4)_ = 25.878, *P* < 0.01]. There was no significant change in *MpGus* expression from the second to the third instar nymph stage (*P* > 0.05). Subsequently, the expression of *MpGus* gradually increased from the third to the fourth instar nymph stage (Figure 6A). Therefore, the expression levels of *MpGus* increased as aphids approached adulthood. The result of *MpGus* expression levels in cephalothorax, legs, and abdomen of apterous adult aphids showed that among the different tissue types, *MpGus* expression was significantly higher in abdomen tissue than in cephalothorax or leg tissue (Figure 6B).

### Expression Levels of *MpGus* Expression Were Down-Regulated by RNAi

**FIGURE 7:**
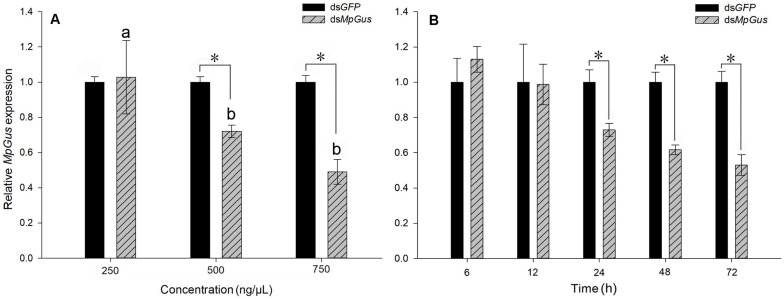
Interference of *MpGus* expression by dsRNA. **(A)** Relative expression levels of *MpGus* fed on different concentrations of ds*MpGus* for 24 h, as measured by RT-qPCR. **(B)** Relative expression levels of *MpGus* fed on 500 ng/μL ds*MpGus* (final concentration) for 6, 12, 24, 48, and 72 h, as measured by RT-qPCR. Each bar represents the mean of three independent samples, each with three repetitions. β*-actin* and *GAPDH* from *M. persicae* were used as reference genes. Values represent mean ± SE, *n* = 3. An asterisk indicates a significant difference (*P* < 0.05) between the expression levels of ds*GFP* and ds*MpGus* at the same concentration. Different letters (a b) above each bar denote significant differences (*P* < 0.05).

In order to determine whether *MpGus* expression levels could be manipulated with RNAi, and whether such manipulation affected the fecundity of *M. persicae* fecundity, we added dsRNA targeting *MpGus* (ds*MpGus*) to an artificial diet for *M. persicae*. The control *M. persicae* group was fed with a non-specific dsRNA based on the green fluorescent protein coding sequence (ds*GFP*). After 24 h of feeding on the artificial diet containing ds*MpGus*, we found that the relative expression levels of *MpGus* did not change in *M. persicae* fed on ds*MpGus* at 250 ng/μL [*F*_(1,4)_ = 0.052, *P* = 0.83 > 0.05]. The expression levels of *MpGus* in adult *M. persicae* were downregulated by 27.9% [*F*_(1,4)_ = 108.708, *P* < 0.05] and 50.9% [*F*_(1,4)_ = 120.661, *P* < 0.05] following treatment with 500 and 750 ng/μL of ds*MpGus*, respectively. However, there was no significant difference between the 500 and 750 ng/μL (Figure 7A). Therefore, our data showed that oral intake of ds*MpGus* was sufficient to cause a significant downregulation of *MpGus* expression in *M. persicae*. According to the experimental results, we selected 500 ng/μL dsRNA as the effective RNAi concentration and further determined the time when *MpGus* expression was significantly downregulated by this concentration. As shown in Figure 7B, the relative expression levels of *MpGus* were downregulated at 12 h but not significantly [*F*_(1,4)_ = 0.502, *P* = 0.518 > 0.05]. *MpGus* expression was significantly downregulated at 24 h [*F*_(1,4)_ = 453.933, *P* < 0.05]. The mortality of *M*. *persicae* was shown in Supplementary Figure S3. There is no significant difference between the control group and the dsRNA treatment group.

### RNAi-Mediated Knockdown of *MpGus* Lowers *M. persicae* Fecundity

**FIGURE 8:**
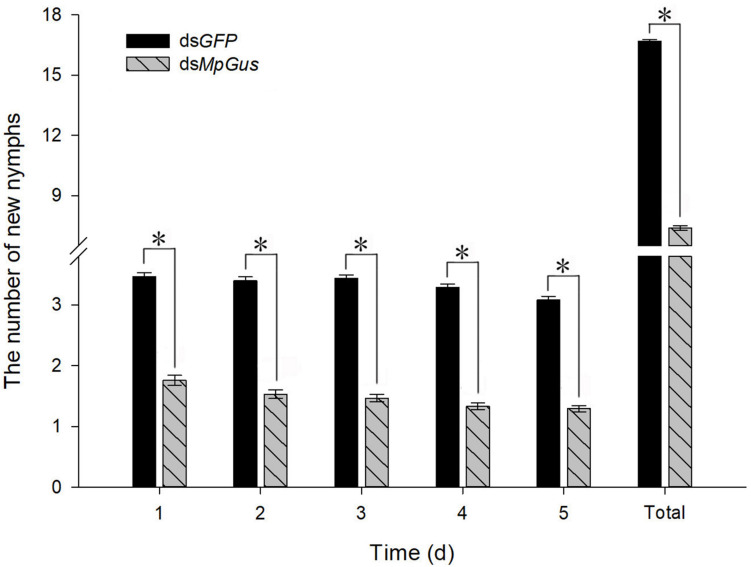
Changes in the fecundity of *M. persicae* in response to intake of dsRNA. Each bar represents the mean number of newborns (± SD) from 75 mother aphids. Counting was carried out for 5 days, with newborns from the previous day removed after each count. An asterisk indicates a significant difference (*P* < 0.05) between aphids fed with ds*GFP* and ds*MpGus*.

Next, we investigated how the knockdown of *MpGus* might affect the fecundity of *M. persicae*. As shown in Figure 7A, an artificial diet containing 500 ng/μL ds*MpGus* was sufficient to cause significant knockdown of *MpGus* mRNA levels in *M. persicae* adults. Next, we investigated *M. persicae* adults with regard to the number of newborn nymphs they produced while fed on 500 ng/μL ds*MpGus*, with those fed with 500 ng/μL of ds*GFP* as controls. As shown in Figure 8, compared with the controls, adults with *MpGus* knockdown produced significantly fewer newborn nymphs. Specifically, the rates of reduction were 49.2% [*F*_(1,148)_ = 267.878, *P* < 0.01], 54.9% [*F*_(1,148)_ = 381.684, *P* < 0.01], 57.4% [*F(*_1,148)_ = 581.800, *P* < 0.01], 59.5% [*F*_(1,148)_ = 661.865, *P* < 0.01], and 58.0% [*F*_(1,148)_ = 477.965, *P* < 0.01] for the first 5 days of *MpGus* knockdown (Figure 8). Collectively, these results strongly suggest that *MpGus* is a positive regulator of fecundity in *M. persicae* and that the expression of *MpGus* at appropriate levels is essential for the reproductive success of this insect.

### Knockdown of *MpGus* Reduced the Number of Developing *M. persicae* Embryos

**FIGURE 9:**
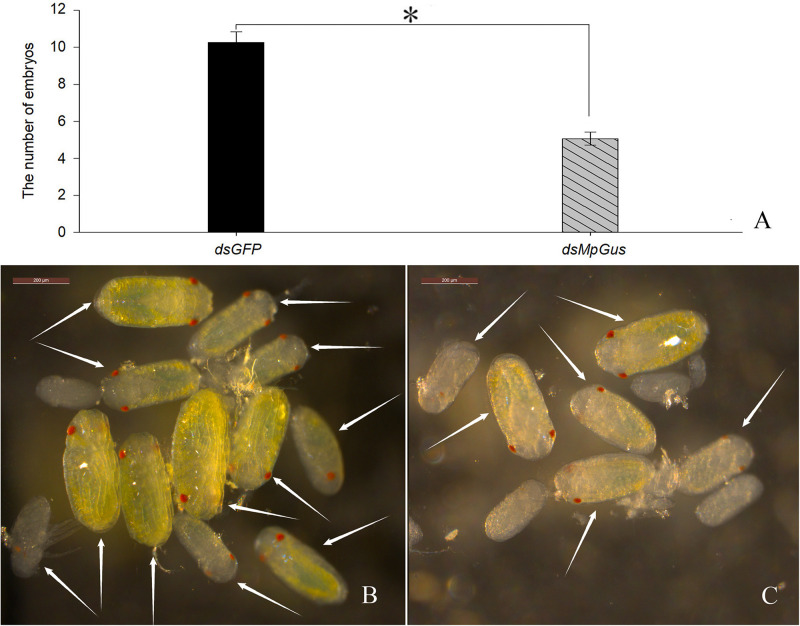
Effect of ds*MpGus* treatment on the number of developing embryos. Counting was carried out on female aphids treated with ds*MpGus* for 24 h. **(A)** Quantitative analysis. Data represent mean ± SD, *n* = 15. Asterisk indicates a significant difference (*P* < 0.05) between ds*GFP* and ds*MpGus*. **(B)** Developing embryos produced by adults treated with ds*GFP*. **(C)** Developing embryos produced by adults treated with ds*MpGus*. Arrows in **(A,B)** indicate embryos with red compound eyes. Scale bars = 200 μm.

In the previous experiment, we found that knockdown of *MpGus* caused *M. persicae* to produce significantly fewer nymphs. Next, we evaluated whether such knockdown also exerted a negative effect on the number of *M. persicae* embryos. As it was difficult to spot embryos in the early stages of development, we only counted developing embryos that had distinct red compound eyes. The results are shown in Figure 9. Compared with the control adults treated with ds*GFP* (Figure 9B), those treated with ds*MpGus* for 24 h showed a notable reduction in the number of embryos produced (Figure 9C). Further quantification using multiple adult aphids indicated that the mean number of developing embryos per aphid treated with ds*MpGus* (5.07) was significantly lower (50.6% reduction, [*F*_(1,28)_ = 59.232, *P* < 0.01] compared with controls (10.27) (Figure 9A). Therefore, *MpGus* appears to have a role that ensures the production of healthy embryos.

### The mRNA Levels of *MpGus* Were Significantly Lower in *M. persicae* Fed on PVY^*N*^-Infected Tobacco Plants

**FIGURE 10:**
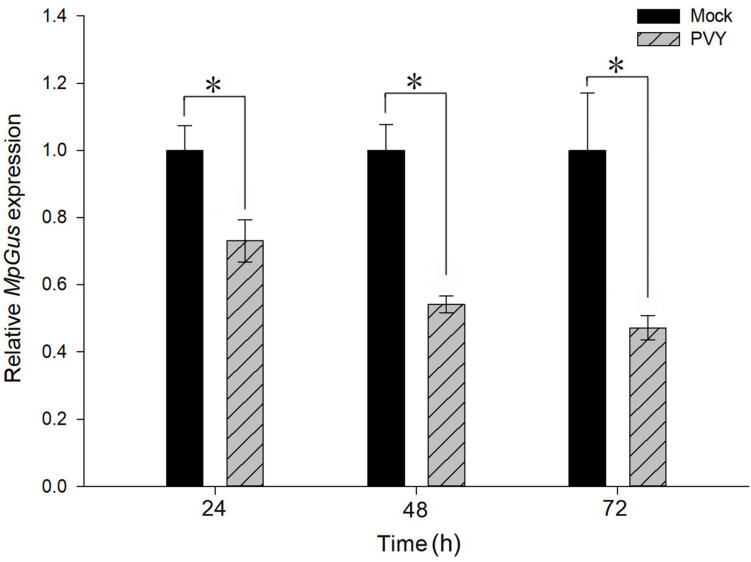
Effects of PVY^*N*^-infected tobacco plants on the relative expression of *MpGus*, as measured by RT-qPCR. Each bar represents the mean of three independent samples, each with three repetitions. β*-actin* and *GAPDH* genes from *M. persicae* were used as references. Values represent mean ± SE, *n* = 3. Asterisk indicates a significant difference (*P* < 0.05).

*M. persicae* is a potent transmission vector for PVY, and the fecundity of *M. persicae* is known to be affected by the acquisition of PVY (Srinivasan and Alvarez, 2007; Ren et al., 2015; Bak et al., 2017). We therefore considered whether feeding *M. persicae* with PVY-infected plants might also perturb the expression of *MpGus*. As shown in Figure 10, levels of *MpGus* mRNA decreased progressively in *M. persicae* fed on PVY^*N*^-infected tobacco plants. Compared with *M. persicae* fed on mock-inoculated tobacco plants, the relative expression levels of *MpGus* in *M. persicae* maintained on PVY^*N*^-infected tobacco plants decreased by 26.9% [*F*_(1,4)_ = 23.129, *P* < 0.05], 45.9% [*F*_(1,4)_ = 96.165, *P* < 0.01], and 52.8% [*F*_(1,4)_ = 27.348, *P* < 0.01] after 24, 48, and 72 h of feeding, respectively.

### *M. persicae* Maintained on PVY^*N*^-Infected Tobacco Plants Produced a Smaller Number of Nymphs

**FIGURE 11:**
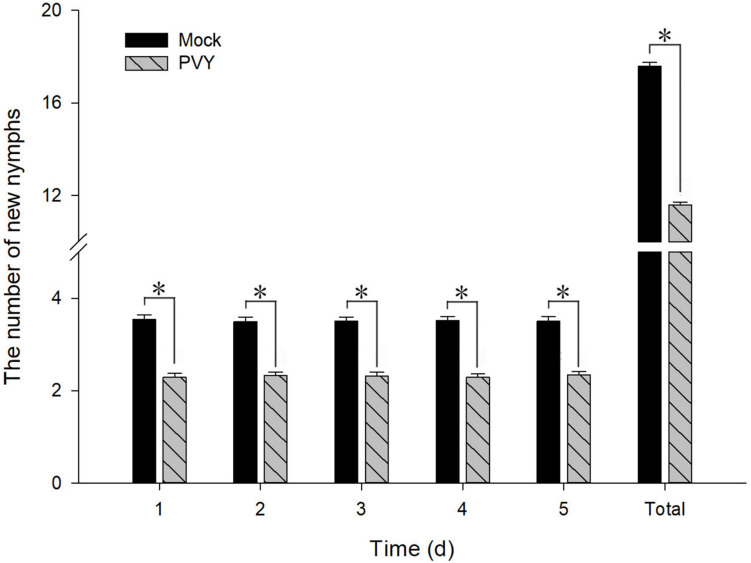
Fecundity of *M. persicae* fed on PVY^*N*^-infected tobacco plants. Newborn nymphs were removed after each count. Values represent mean ± SD. Asterisk indicates a significant difference (*P* < 0.05).

To determine the effect of PVY^*N*^ on the fecundity of *M. persicae*, we counted the numbers of newborn nymphs produced by adult aphids fed on PVY^*N*^-infected tobacco plants over a period of 5 days. As shown in Figure 11, both the daily means and the total number of nymphs counted over the 5 days were significantly lower when compared with controls. The mean number of newborn nymphs produced by *M. persicae* maintained on PVY^*N*^-infected tobacco plants decreased by 35.3% [*F*_(1,148)_ = 94.626, *P* < 0.01] within 24 h of feeding. However, the level of reduction did not appear to intensify thereafter, as the rate of reduction remained relatively stable at 33.2% [*F*_(1,148)_ = 91.730, *P* < 0.01], 33.8% [*F*_(1,148)_ = 104.113, *P* < 0.01], 34.8% [*F*_(1,148)_ = 118.849, *P* < 0.01], and 33.1% [*F*_(1,148)_ = 93.663, *P* < 0.01], over the subsequent 4 days. Collectively, these data suggest that PVY^*N*^-infected tobacco plants constituted a hostile environment for *M. persicae* reproduction, possibly forcing the aphids to migrate to virus-free tobacco, thus facilitating viral transmission. Importantly, the virus-infected plants appear to exert this effect by down-regulating the expression of *MpGus*, thus highlighting an indispensable role for *MpGus* in the reproduction of *M. persica*e.

## Discussion

Gustavus is a protein that was first identified in *D. melanogaster* and found to play an essential part in ensuring the maturation of germline cells (Styhler et al., 2002). A Gus ortholog has since been characterized in a distantly related arthropod (*M. nipponense*) and was shown to have a strikingly similar reproduction-related role (Zhang et al., 2011). In the present study, we aimed to identify a candidate gene in *M. persicae* that could be targeted with RNAi to facilitate the control of this agricultural pest. Here, we report the successful identification and preliminary characterization of the *Gus* ortholog of *M. persicae*, designated *MpGus*. This represents the first identification and characterization of a *Gus* ortholog in an aphid species.

Despite the relatively distant phylogenetic relationship, the MpGus protein shares the highly conserved SPRY domain and SOCS box with its orthologs in *D. melanogaster* (Styhler et al., 2002) and other insects. These findings were consistent with those reported previously by Xing et al. (2006). Our study showed that about 90 amino acids, located in the N-terminal of the MpGus protein prior to the SPRY domain and SOCS box, were highly conserved in aphid species compared with other species (Figure 4). This species-specific conserved sequence provides us with the possibility to solve the safety problems of RNAi off-target effects on non-target organisms (Roberts et al., 2015). This also suggested that RNAi preparations for *MpGus* could be used to target a variety of aphids, such as *A. gossypii*, *R. maidis*, *M. sacchari*, *D. noxia*, and *A. pisum*. Furthermore, it suggested that MpGus and its ortholog proteins have important biological functions in aphids.

The expression patterns in different tissues demonstrated that *MpGus* was highly expressed in the abdomens of apterous adult aphids, suggesting a potential role in reproduction. The expression pattern in different instar nymphs showed that the expression of *MpGus* gradually increased as aphids aged. It has been reported that embryos are present in all developmental stages of aphids, and that mature embryos already existed in nymphs from the fourth instar nymph stage (Xu and Wu, 2020). Styhler et al. (2002) confirmed that the expression of *Gus* was related to both ovarian and embryonic development in *D. melanogaster*. Therefore, an increased level of *MpGus* expression might be related to the development of embryos as well as to reproduction. In addition, the SPRY domain could mediate the release of Ca^2+^ in the sarcoplasmic reticulum (Ponting, 1997). Zhang et al. (2011) found that the *Gus* ortholog of *M. nipponense* is highly expressed in muscle tissue. This suggested that *MpGus* may also be involved in the development of muscle tissue in nymphs. To further investigate the effect of *MpGus* on reproduction, we performed RNAi-mediated knockdown experiments on adult aphids.

Our results demonstrated that feeding on 500 ng/μL of ds*MpGus* for 24 h can significantly reduce the expression of *MpGus*. We successfully knocked down *MpGus* expression by feeding adult *M. persicae*; this resulted in a significant reduction in the number of nymphs produced by these adults. These results further suggest that *MpGus* regulates the fecundity of *M. persicae*; these findings are consistent with those of previous studies in *D. melanogaster* (Styhler et al., 2002; Kugler et al., 2010).

To understand how *MpGus* affects fecundity, we evaluated the development of embryos in adult *M. persicae* following the knockdown of *MpGus*. Similar methods have been used in previous studies to confirm the effects of genes or other factors on the fecundity of aphids (Shang et al., 2017; Xu and Wu, 2020). Our results demonstrated that the RNAi-mediated knockdown of *MpGus* caused a significant reduction in the number of developing embryos in *M. persicae* compared with the number of embryos produced by the control group. This reduction in the number of mature embryos explains the decline in fecundity observed in adult aphids affected by ds*MpGus*. Thus, we speculated that downregulation of *MpGus* expression affects the processes of embryonic development and maturation, and ultimately reduces the fecundity of *M. persicae*.

We also found that fecundity was reduced when we fed *M. persicae* on PVY^*N*^-infected tobacco plants. However, our results appear to contradict previous reports by Srinivasan and Alvarez (2007) and Bak et al. (2017), which stated that PVY-infected plants exerted a positive effect on the fecundity of *M. persicae*. This discrepancy could be attributed to a variety of factors, including viral strains, host plants, *M. persicae* isolates, and environmental conditions (Ren et al., 2015). Nevertheless, it is notable that the virus-associated reduction in *M. persicae* fecundity observed in our study was accompanied by lower expression levels of *MpGu*s; this suggests that *MpGus* plays a role in the regulation of *M. persicae* fecundity by PVY^*N*^-infected plants.

In the experiments described herein, we used home-made mini cages to monitor the daily reproduction of a single aphid maintained on a known plant or artificial diet. This form of investigation has not been described previously. In previous studies, experiments were carried out using a clip cage (2–2.5 cm in diameter) (Srinivasan and Alvarez, 2007; Kersch-Becker and Thaler, 2013; Bak et al., 2017). However, owing to their excessive weight and volume, only 1–2 clip cages can be placed on each leaflet, thus leading to a smaller sample size or the need for a larger number of plants (Srinivasan and Alvarez, 2007; Ren et al., 2015). By contrast, using home-made mini cages, we were able to acquire data from 75 individual aphids on a single tobacco plant in the present study. This strategy helped to reduce statistical error caused by differences between different plants, significantly improved the efficiency of our research, and meant that we could collect data from a larger sample size, thus improving the accuracy of our quantitation. This approach is particularly useful when a large sample size of diverse insects, such as whitefly, thrips, and aphids, is required.

In summary, we identified and characterized the *Gus* gene in *M. persicae* (*MpGus*) and found that *MpGus* has a critical role in the reproductive cycle of *M. persicae*. Our results also suggest that *MpGus* is involved in the effect of PVY^*N*^ on the fecundity of *M. persicae*. *MpGus* may also affect the development and survival of nymphs, but this needs further study. In this study, the downregulation of *MpGus* inhibited embryo development and reduced fecundity in *M. persicae*, thus providing evidence that *MpGus* could be used as a target for the control of *M. persicae*. Consequently, our findings indicate that *MpGus* is a new and effective target for the control of *M. persicae*.

## Data Availability Statement

The datasets presented in this study can be found in online repositories. The names of the repository/repositories and accession number(s) can be found in the article/Supplementary Material.

## Author Contributions

YL, LD, and DZ designed the research. YG and RR conceived the experiments. YG, JP, and DW analyzed the data. YG drafted the manuscript. XS, LZ, ZZ, CZ, YL, LD, and DZ revised and finalized the manuscript. All authors contributed to the article and approved the submitted version.

## Conflict of Interest

The authors declare that the research was conducted in the absence of any commercial or financial relationships that could be construed as a potential conflict of interest.

## Footnotes

1 http://www.ncbi.nlm.nih.gov/BLAST/

2 https://www.ebi.ac.uk/Tools/msa/clustalo/

3 http://www.megasoftware.net/

## Supplementary Material

Supplementary Figure 1 Sequence alignment and electrophoresis detection of PCR product of primer RT-qPCR-MpGus-F/R. **(A)** Two-sequence alignment between PCR product and MpGus-X3. **(B)** Agar gelatin electrophoresis result of PCR product.

Supplementary Figure 2 Amplification specificity and amplification efficiency of primer RT-qPCR-MpGus-F/R in RT-qPCR. **(A)** Melting curve of RT-qPCR product. **(B)** Standard curve.

Supplementary Figure 3 Effect of dsRNA on *M. persicae* mortality. Each group contained 75 aphids. Counting was carried out for 5 days. Data represent mean ± SD, *n* = 3. There was no significant difference in mortality among the three groups at each counting point.
