# Supplementary figures and images for: The Gustavus Gene Can Regulate the Fecundity of the Green Peach Aphid, Myzus persicae (Sulzer)
Source: Front Physiol. 2021 Jan 12;11:596392. doi: 10.3389/fphys.2020.596392 (PMC7835840; doi:10.3389/fphys.2020.596392)

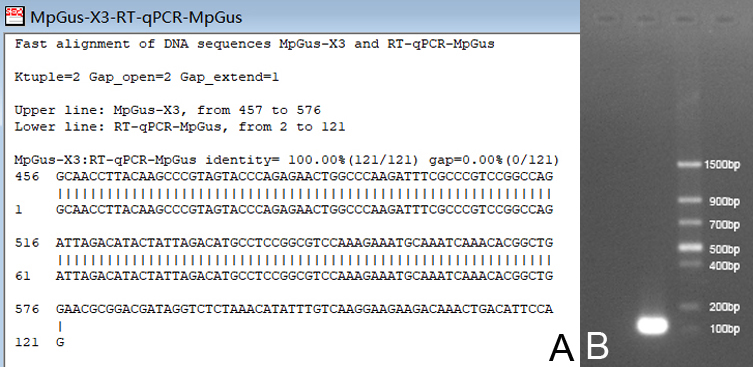

Supplement: Supplementary Figure 1 — Sequence alignment and electrophoresis detection of PCR product of primer RT-qPCR-MpGus-F/R. (A) Two-sequence alignment between PCR product and MpGus-X3. (B) Agar gelatin electrophoresis result of PCR product. [file Figure_1.JPEG]

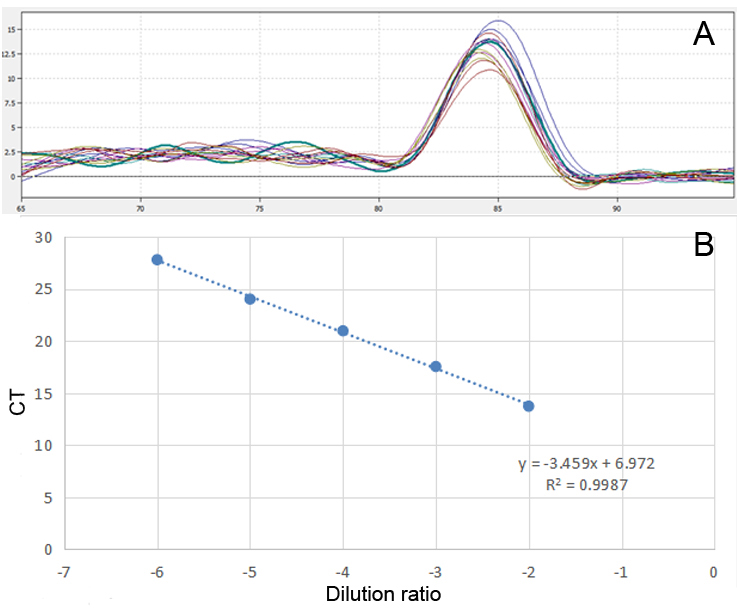

Supplement: Supplementary Figure 2 — Amplification specificity and amplification efficiency of primer RT-qPCR-MpGus-F/R in RT-qPCR. (A) Melting curve of RT-qPCR product. (B) Standard curve. [file Figure_2.JPEG]

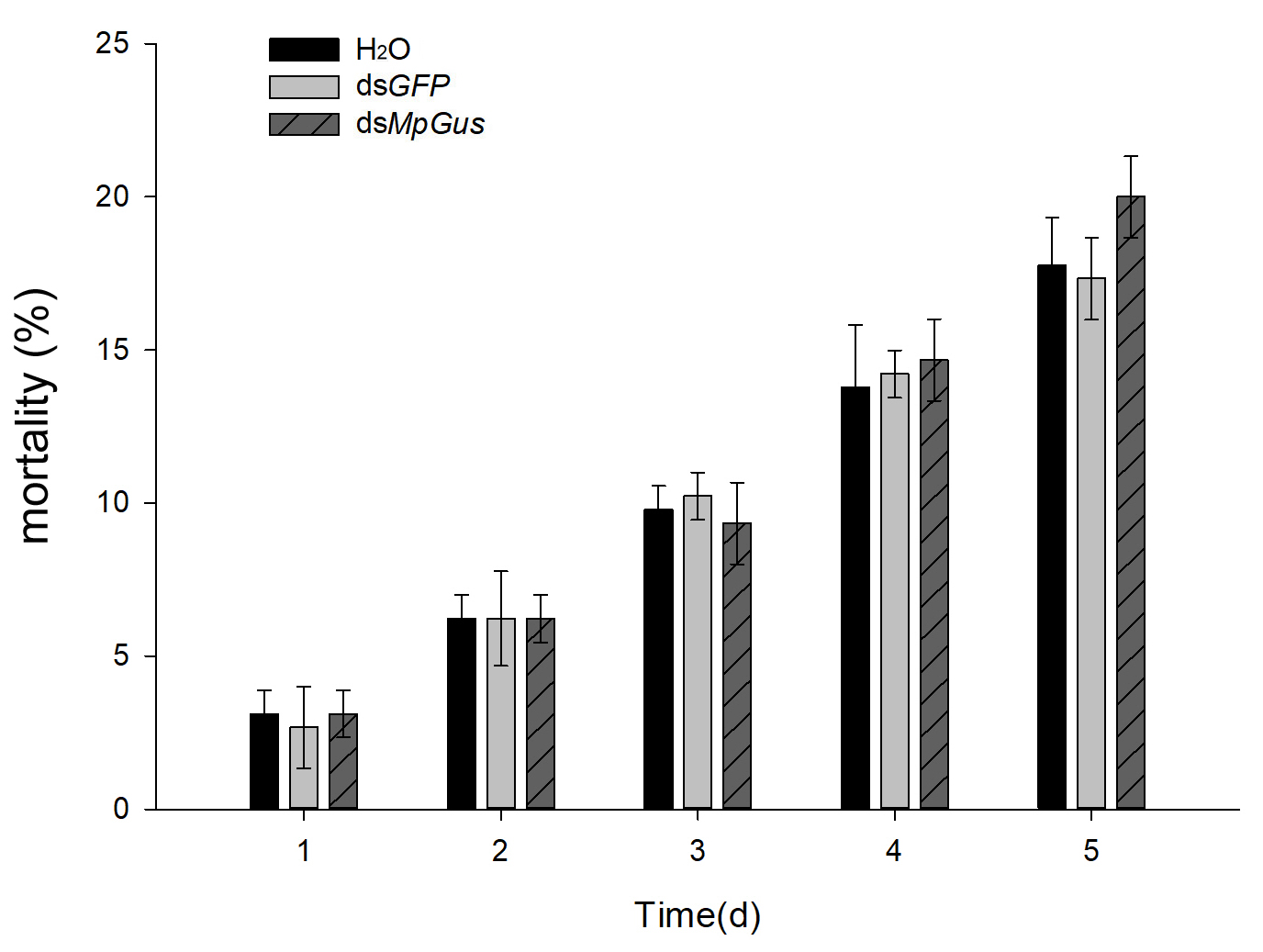

Supplement: Supplementary Figure 3 — Effect of dsRNA on M. persicae mortality. Each group contained 75 aphids. Counting was carried out for 5 days. Data represent mean ± SD, n = 3. There was no significant difference in mortality among the three groups at each counting point. [file Figure_3.JPEG]
